# Aniridia-related keratopathy: Structural changes in naïve and transplanted corneal buttons

**DOI:** 10.1371/journal.pone.0198822

**Published:** 2018-06-11

**Authors:** André Vicente, Berit Byström, Mona Lindström, Ulf Stenevi, Fátima Pedrosa Domellöf

**Affiliations:** 1 Department of Clinical Science, Ophthalmology, Umeå University, Umeå, Sweden; 2 Department of Integrative Medical Biology, Section for Anatomy, Umeå University, Umeå, Sweden; 3 Department of Ophthalmology, University of Gothenburg, Gothenburg, Sweden; Cedars-Sinai Medical Center, UNITED STATES

## Abstract

**Background:**

To study structural changes in naïve and surgically treated corneas of aniridia patients with advanced aniridia-related keratopathy (ARK).

**Methods and findings:**

Two naïve corneal buttons from patients with advanced ARK submitted to penetrating keratoplasty for the first time, one corneal button from an ARK patient that had undergone a keratolimbal allograft (KLAL), two corneal buttons from ARK patients who had previously undergone centered or decentered transplantation and were now retransplanted and two adult healthy donor control corneas were processed for immunohistochemistry. Antibodies against extracellular matrix components in the stroma and in the epithelial basement membrane (collagen I and IV, collagen receptor α11 integrin and laminin α3 chain), markers of fibrosis, wound healing and vascularization (fibronectin, tenascin-C, vimentin, α-SMA and caveolin-1), cell division (Ki-67) and macrophages (CD68) were used. Naïve ARK, KLAL ARK corneas and transplanted corneal buttons presented similar histopathological changes with irregular epithelium and disruption or absence of epithelial basal membrane. There was a loss of the orderly pattern of collagen lamellae and absence of collagen I in all ARK corneas. Vascularization was revealed by the presence of caveolin-1 and collagen IV in the pannus of all ARK aniridia corneas. The changes observed in decentered and centered transplants were analogous.

**Conclusions:**

Given the similar pathological features of all cases, conditions inherent to the host seem to play an important role on the pathophysiology of the ARK in the long run.

## Introduction

Aniridia is a rare disorder caused by mutations in the PAX6 gene, essential for the development of the eye. The age-specific prevalence in a Swedish cohort of cases < 20 years of age is 1:47000 [1]. The developmental disturbances associated with aniridia affect several different structures of the eye, including the cornea, anterior chamber, iris, lens, retina and optic nerve [2] and one of the most visually significant consequences is aniridia related keratopathy (ARK).

ARK usually includes limbal stem cell-deficiency associated with impaired epithelial cell adhesion, conjunctivalization and corneal vascular pannus, that typically appear after childhood [2, 3, 4] and result in a thickened vascularized cornea prone to repeated erosions with chronic irritation and vision-threatening opacification [5, 6]. Aniridia is often difficult to manage both medically and surgically. Cataract and glaucoma surgery as well as corneal surgery can accelerate ARK [7, 8]. In early stages of ARK, quiescent epithelial inflammation has been detected with in vivo confocal microscopy in corneas that are still clear [9, 10].

Cornea surgery in aniridia patients has a limited prognosis and is therefore avoided as far as possible. Because of poor healing response and limbal stem cell deficiency, penetrating keratoplasty has a poor prognosis in aniridia corneas. When vision is severely impaired and medical treatment is no longer enough, limbo-keratoplasty appears to be a better treatment alternative in ARK [5, 11]. However, these patients need systemic immunosuppression to achieve ocular surface stability and improved visual acuity [11]. Another treatment option available for ARK patients is keratolimbal allograft transplantation (KLAL). Tsubota and co-workers [12] were the first to describe the use of stored corneoscleral rim for limbal stem cell transplantation and later, this technique was modified to include two corneoscleral rims [13]. The anterior stromal and epithelial portion of the harvested limbal rims from an allogeneic donor are transplanted in two to three 120°-180° segments to the recipient limbus after limbal pannus/stromal dissection, without manipulation of the central cornea, in order to completely surround the patient´s limbus [13, 14]. The main difference between KLAL and limbo-keratoplasty is that in KLAL, there is no interference with the central cornea of the patient. The majority of patients successfully operated with this technique required triple immunosuppressive therapy [15]. KLAL does not deliver a source for conjunctival tissue and is, hence, a procedure of choice when there is primary limbal involvement with minor conjunctival changes, such as in patients with ARK [16].

Because of the restrictions of surgery, full thickness corneal samples of eyes with aniridia are extremely rare. In the present study, we investigated the full thickness of two corneal buttons from patients with advanced aniridia submitted to penetrating keratoplasty for the first time (herein referred to as naïve ARK corneas, as they were not previously submitted to keratoplasty), one corneal button from a patient that had previously undergone a KLAL and was now submitted to a centered transplantation (herein referred to as KLAL ARK cornea) and two corneal buttons from aniridia patients with advanced keratopathy that had undergone centered or decentered transplantation. That is, we studied the donor corneas that had acquired ARK features after transplantation to aniridia patients (herein referred to as transplanted ARK corneas). The evaluation of the histopathological patterns that occur in naïve ARK, KLAL ARK, centered and decentered transplanted ARK corneas make the present study unique and may provide valuable insights into the pathophysiology of ARK. The samples were studied with immunohistochemistry using a large battery of antibodies against extracellular matrix components in the stroma and the epithelial basement membrane such as collagen I and IV, collagen receptor α11 integrin, and laminin α3 chain; markers of fibrosis, wound healing and vascularization (fibronectin, tenascin-C, vimentin, α-SMA and caveolin-1), cell division (Ki-67) and macrophages (CD68).

Corneal transparency is highly dependable on the precise organization of stromal collagen fibrils [17, 18] and therefore we investigated collagen type I (the main type responsible for stromal structure and function), and collagen type IV (a network-forming collagen in basement membranes) [19]. The collagen receptor α11 integrin, a mediator in cell adhesion to collagen I, IV and V, has been associated with the organization of the corneal collagen matrix as cell-collagen interactions are crucial for the reorganization of the collagen matrix in both wound healing and developing tissues [20, 21]. Furthermore, α11 integrin chain has been suggested to play a role in collagen deposition in keratoconus related corneal scarring and in human corneal development [21]. Matrix markers of fibrosis and wound healing such as fibronectin and tenascin-C, that are temporally upregulated in injured mesenchymal tissue, were also evaluated. Tenascin-C modulates growth factor signaling in macrophages and mesenchymal cells in the corneal stroma and influences, therefore, corneal wound healing processes as it regulates inflammation, neovascularization and fibrosis [22]. It has been proven that the presence of macrophages and myofibroblasts, an essential step for primary healing or excess fibrosis after stromal corneal injuries, is significantly downregulated in a tenascin-C null mouse model [23].

Alpha smooth muscle actin (α-SMA) is a protein related to cell motility, structure and integrity, commonly used as a marker of myofibroblast formation [24]. Vimentin is an intermediate filament protein present at low levels in normal keratocytes, but overexpressed in the corneal stroma after injury, as it provides structural force essential for wound contracture [25].

Caveolin-1 has been proposed as a regulator of corneal epithelial wound-healing capacity and is also present in the caveolae membranes of blood vessels. It is involved in growth-factor induced cellular signal transduction [26]. Ki-67, a cell division marker, laminin α3 chain, a major non-collagenous component of basement membranes and the macrophage marker CD68 were also evaluated.

Our study is the first to compare the histopathological changes in naïve ARK corneas (the patient’s own cornea button removed at the time of the first transplantation) and an ARK cornea after a keratolimbal allograft (corneal button obtained during a keratoplasty after a previous KLAL procedure that failed) with transplanted ARK corneas (from patients who were retransplanted due to failure with a former centered or decentered transplant with a donor cornea from a healthy donor). Our data indicate that there is a similar derangement of stromal architecture with a subepithelial pannus containing abnormal molecules replacing the normal collagen I lamellae in naïve ARK corneas, KLAL ARK corneas and transplanted ARK corneas, suggesting that a pathological corneal microenvironment of the host is likely to play a key role.

## Methods

The present study comprised two naïve corneal buttons from patients with advanced ARK submitted to penetrating keratoplasty for the first time (cases A and B), one corneal button from an ARK patient that had undergone a KLAL (case C), two corneal buttons from ARK patients who had previously undergone centered (case D) or decentered (case E) transplantation and were now retransplanted and two adult healthy donor control corneas; one (74-year-old male, who had previously undergone cataract surgery), obtained immediately after evisceration due to an ocular tumor, and another (82-year-old male, who had not undergone any ocular surgery) obtained from a donor who had chosen, when alive, to donate his corneas for research post-mortem. This control cornea was collected postmortem within the time frame of the European guidelines for tissue use for human transplantation, guaranteeing the viability of the tissue samples. Control corneas were not age-matched with the patients. However, it is well known that corneas normally maintain their transparency and structure throughout life, irrespective of age and that age does not affect the viability of corneal buttons for transplantation [27, 28]. The diagnosis of aniridia was based on typical clinical characteristics [5, 29] and genetic analysis had also been performed in cases D and E and identified a heterozygous mutation in the PAX6 gene.

**Fig 1:**
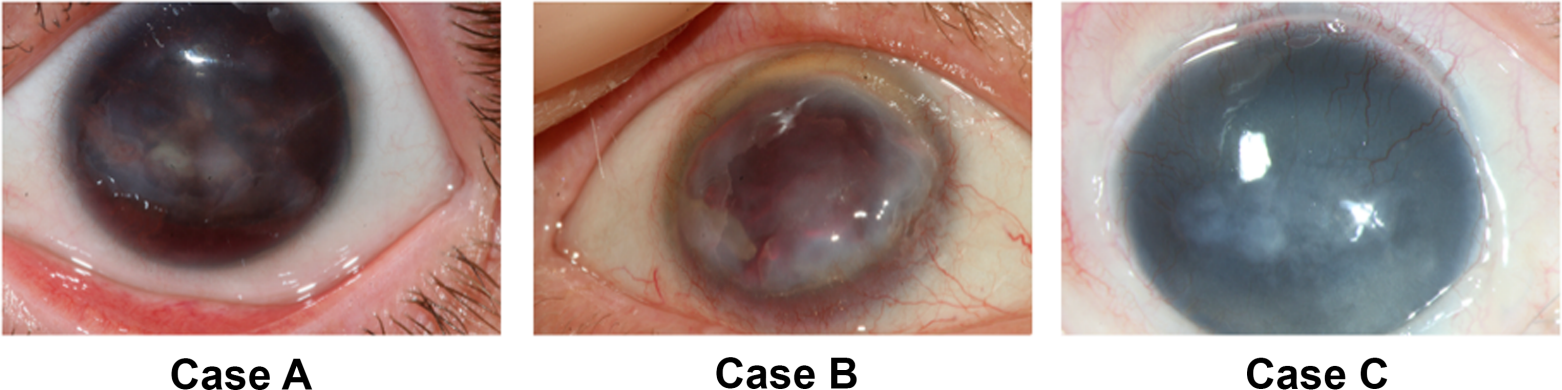
**Preoperative photographs of the corneas of three aniridia patients (cases A, B and C).** Notice the cloudy corneas with the absence of normal red retinal reflex due to the opaque ARK and the ingrowth of blood vessels from the conjunctiva into the corneas, best seen in the superior part of the cornea in case C.

Case A (Fig 1) was a 29-year old woman with vision less than 20/200 and an opaque cornea with irregular epithelium with recurrent erosions. The recurrent erosions and corneal opacities were unresponsive to milder surgical procedures (corneal epithelial debridement). The patient did not agree to keratolimbal stem cell transplantation because she did not want systemic immunosuppression. A centered penetrating keratoplasty was performed instead, even though a guarded prognosis was expected with high risk for failure with pannus overgrowth, in order to address the recurrent erosions. Case B (Fig 1) was a 65-year old man who earlier had cataract extraction with resulting aphakia. He had previously undergone a phototherapeutic keratectomy because of recurrent corneal erosions but with no positive effect. The cornea remained cloudy and the corneal surface problems were severe. Immunosuppression was not a possible alternative due to his overall health condition and therefore a Boston keratoprosthesis was implanted following the penetrating keratoplasty. Case C (Fig 1) was a 38-year old female who had had a KLAL transplantation, according to the technique described by Holland et *al*. (2003), two years earlier but the cornea did not clear, cataract developed, and the visual acuity remained low. Due to the almost opaque cornea and the dense cataract, she underwent a centered penetrating keratoplasty combined with cataract extraction. Case D was a 35-year old woman who was submitted to a centered retransplantation after a first centered corneal transplantation due to recurrence of ARK with persistent corneal erosions and severe ocular surface disease. Case E was a 37-year old woman who underwent a second decentered retransplantation after severe ocular surface problems with recurrent corneal erosions and persistent cloudiness of the first decentered transplanted cornea.

The samples were collected with the approval of the Regional Ethical Commitee, Umeå and Gothenburg following informed consent and in accordance with the tenets of the declaration of Helsinki. The corneal buttons were routinely formalin-fixed, embedded into paraffin wax and serial sections, 4 μm thick, were collected on Superfrost® Plus slides (Thermo scientific), dried in a vertical position overnight at 60°C and thereafter stored at +4°C in a tightly closed slide box until further processing. Two normal corneas were processed and sectioned using the same protocol.

The corneal sections were treated as previously described [30]. In summary, sections were de-waxed in Tissue clear® (1466, Sakura) and rehydrated. Antigen retrieval was performed in a water bath at 95°C for 30 min in pre-warmed citrate buffer (10mM Citric acid, 0,05% Tween 20, pH 6,0) or Tris-EDTA buffer (10mM Tris Base, 1mM EDTA, 0,05% Tween 20, pH 9.0) and allowed to cool down at room temperature for 20 min before the slides were rinsed in running water for 5 min and put into 0,01M phosphate buffered saline (PBS). Unspecific binding of secondary antibodies was blocked with 5% normal donkey or goat serum at room temperature for 15 min prior to the incubations with primary and secondary abs. Primary abs (Table 1) and secondary abs (Table 2), were applied and the sections were incubated at +4°C over night and at 37°C for 30 min, respectively, and washed three times for 5 min in 0,01M PBS containing 0,01% NaN3. The sections were finally mounted in Vectashield® mounting media with 4’,6-diamidino-2-phenylindole (DAPI) (H-1200, Vector laboratories, Inc, Burlingame, USA) for visualization of nuclei.

**Table 1:** Primary antibodies.

Antigen	Antibody	Concentration	Source
Collagen type I	GTX 26308	1:5000	GeneTex, Inc., Irvine, USA
Collagen type IV	M0785	1:500	DakoCytomation, Glostrup, Dk
α11 integrin	α11 integrin	1:8000	Velling et al., 1999
α-SMA	M0851	1:500	DakoCytomation, Glostrup, Dk
Laminin α3	BM-2	1:1000	Rousselle et al., 1991
Fibronectin	A0245	1:1000	DakoCytomation, Glostrup, Dk
Tenascin-C	4A10	1:10	Gullberg et al., 1997
Vimentin	M0725	1:1000	DakoCytomation, Glostrup, Dk
Ki-67	NCL-Ki-67p	1:10000	Novocastra, Leica Biosystems, Newcastle, UK
Caveolin-1	ab2910	1:7500	Abcam, Cambridge, UK
CD68	M0814	1:1000	DakoCytomation, Glostrup, Dk

**Table 2:** Antibodies.

Antigen	Antibody	Concentration	Source
Donkey anti-mouse DyLight 488	715-485-150	1:100	Jackson ImmunoResearch Europe Ltd, Suffolk, UK
Donkey anti-rabbit FITC	715-095-152	1:50	Jackson ImmunoResearch Europe Ltd, Suffolk, UK
Goat anti-rabbit Alexa 488	A11034	1:300	Molecular Probes, Life Technologies, Darmstadt, Germany
Goat anti-mouse Alexa 488	A21121	1:300	Molecular Probes, Life Technologies, Darmstadt, Germany

Well-characterized monoclonal (mAB) or polyclonal (pAB) primary antibodies against collagen type I and type IV; α11 integrin; laminin α3 chain; vimentin; tenascin-C; fibronectin, CD68; caveolin-1; Ki-67 and α-SMA were used (Table 1). The primary antibody was omitted in control sections to reveal potential unspecific binding. Hematoxylin-eosin sections were also prepared for evaluation of general morphology.

Our previous work on human corneas using most of the antibodies above was performed on freshly frozen specimens [21, 31, 32]. In order to be able to appropriately evaluate the staining patterns obtained here in formalin fixed specimens we divided an additional cornea in two halves. One half was freshly frozen and the other half was formalin fixed and processed for paraffin sections as described above. After the immunohistochemical treatment described above, we compared the staining patterns in the two halves of the same cornea and found equal patterns of immunoreactivity for each antibody used. Unfortunately, in our hands, additional antibodies against collagen type III an V did not work in the paraffin sections.

## Results

**Fig 2:**
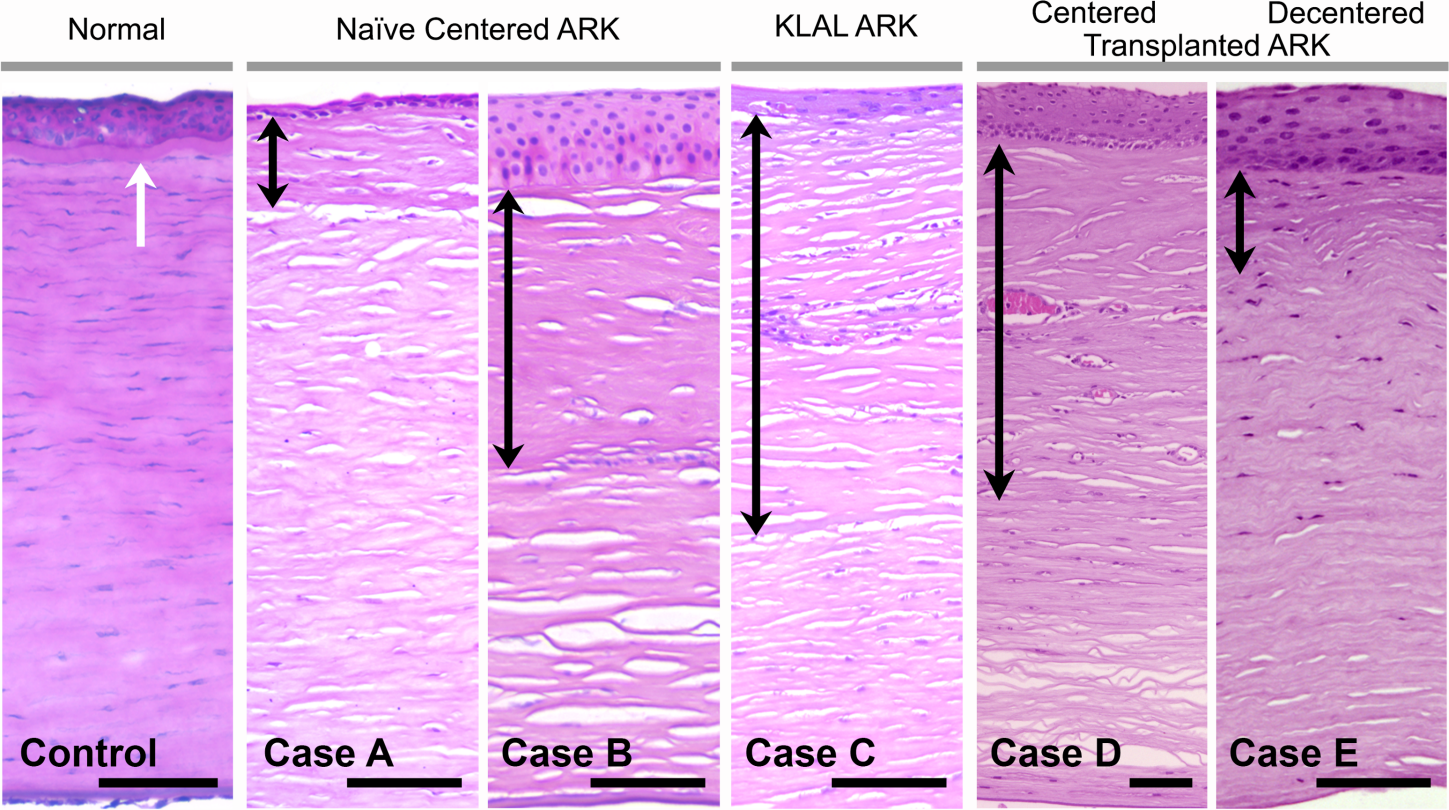
**Cross-sections of a normal adult human cornea (to the left) and corneas from the five aniridia cases (naïve ARK, cases A-B; KLAL ARK, case C; centered transplanted ARK, case D; decentered transplanted ARK, case E) stained with hematoxylin & eosin.** There was considerable variation in epithelium thickness (shown at the top of the photos above the black arrows in A-E), in the aniridia cases. The normal cornea had a clearly visible Bowman’s layer (white arrow) whereas in both the naïve ARK corneas, KLAL ARK cornea and transplanted ARK corneas the epithelial basement membrane appeared to be absent or disrupted and the Bowman’s layer could not be identified. Instead, there was a thick subepithelial pannus in the anterior stroma (black double headed arrows in cases A-E), comprising approximately half of the stroma in the central part of cases B, C and D, and less than 50% in case A and E. Bars 100 μm.

All five aniridia corneas had a rather similar pathological appearance in hematoxylin-eosin sections (Fig 2A–2E). In both naïve ARK, KLAL ARK and transplanted ARK corneas, the epithelium was irregular with large variation in cell size and form and with varying thickness throughout consecutive sections, in contrast to the epithelium with regular thickness and morphology present in control corneas (Fig 2). The epithelial basement membrane of all aniridia corneas was fragmented and appeared to be absent in long segments in which the Bowman’s layer could not be identified (Fig 2). A thick subepithelial pannus was present in the anterior stroma (Fig 2A–2E, arrows). In the two naïve ARK corneas, the pannus comprised less than half of the central corneal thickness in case A and approximately half of the thickness in case B (Fig 2). In the KLAL ARK (case C) and in the centered transplanted ARK (case D) corneas, the subepithelial pannus accounted for approximately half of the corneal thickness. In the decentered transplanted ARK cornea (case E) it comprised less than half of the corneal thickness (Fig 2). Importantly, the subepithelial pannus was also present in parts of the periphery of corneal buttons of both naïve ARK, KLAL ARK and transplanted ARK corneas. The endothelium and Descemet´s membrane appeared normal in all ARK corneas.

**Fig 3:**
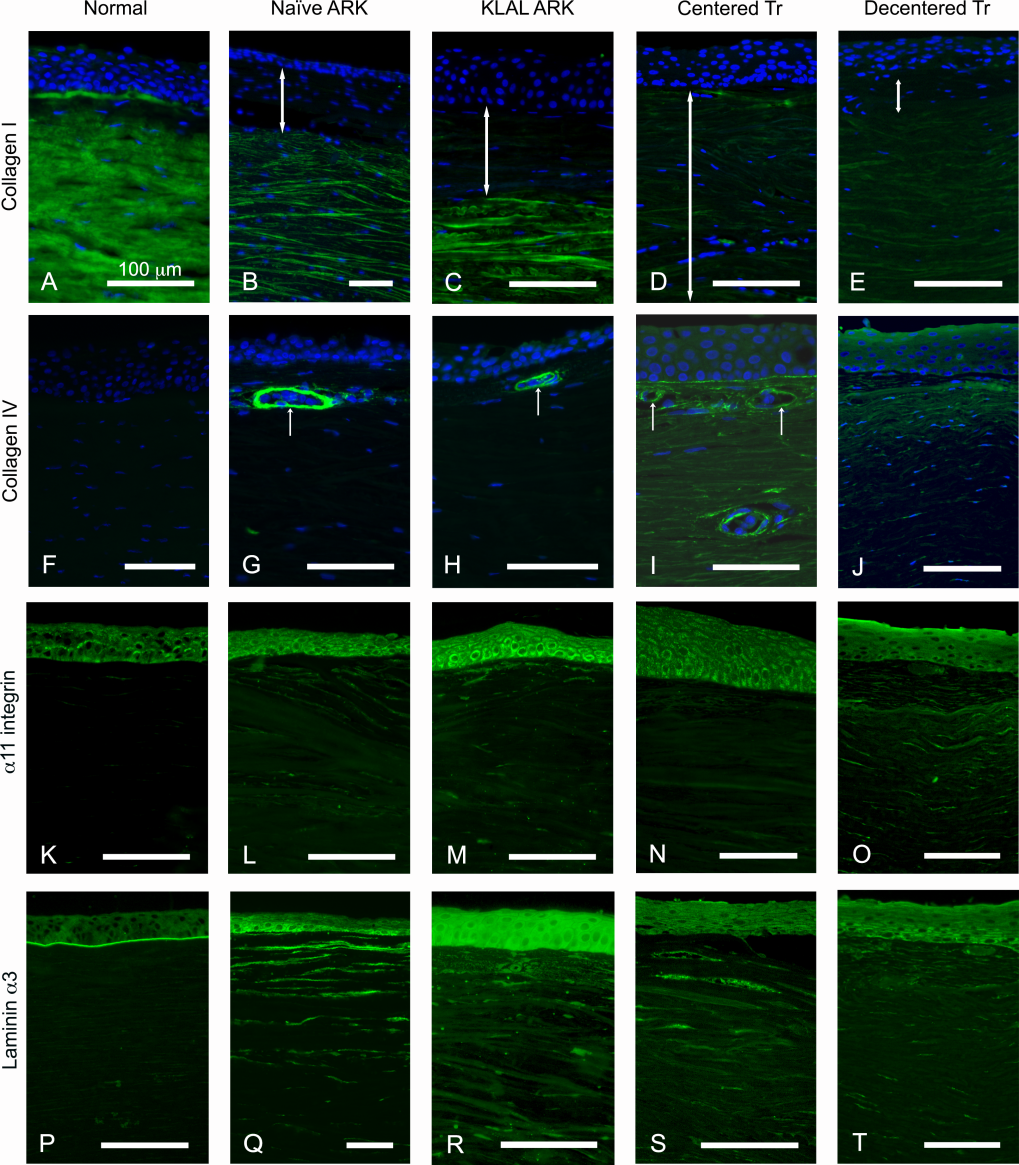
**Normal (A, F, K and P), naïve ARK corneas (B, G, L, Q), KLAL ARK cornea (C, H, M, R), centered transplanted ARK cornea (D, I, N, S) and decentered transplanted ARK cornea (E, J, O, T) labeled with antibodies (green) against collagen I (A-E), collagen IV (F-J),** α**11 integrin (K-O) and laminin** α**3 chain (P-T) and with nuclear labeling with DAPI (blue, A-J).** Notice the presence of collagen I in the normal cornea (A), whereas in the aniridia cases the subepithelial pannus (B-E, white double arrows) lacked collagen I. Collagen IV was identified in the subepithelial pannus in all ARK corneas, although it was weakly labelled in naïve ARK corneas (G) and the KLAL ARK cornea (H), and strongly in the centered transplanted ARK cornea (I) and decentered transplanted ARK cornea (J). Collagen IV was also detected in blood vessels of ARK corneas (G-I) but was absent in control corneas (F). Anti α11 integrin, a receptor for collagen I, labeled the contour of epithelial cells in the normal cornea (K), as previously reported, and in the aniridia cases (L-O). There was also an irregular staining pattern in the anterior stroma (pannus) with anti α11 integrin in the ARK cases (L-O). Laminin α3 chain was present in the basement membrane of the normal cornea (P). In the ARK corneas (Q-T) it was also present in the fragmented epithelial basement membrane and additionally in streaks in the subepithelial pannus. The epithelial basement membrane was apparently absent in large portions of all ARK corneas, as shown in S-T. Bars 100 μm.

The normal corneal stroma constitutes 90% of the corneal thickness and is predominantly composed of collagen type I. The antibody against collagen I strongly labelled the stroma in the control corneas (Fig 3A). In both naïve, KLAL and transplanted ARK corneas (cases A-E), the anterior stroma occupied by the subepithelial pannus either lacked collagen I or was only faintly labelled (Fig 3B–3E) and contained abundant cells revealed by DAPI-staining, whereas the remaining stroma had an almost normal morphology and was labelled with the antibody against collagen type I.

The subepithelial pannus was labelled by the antibody against collagen IV, in all ARK corneas, although it was weakly labelled in naïve ARK corneas (Fig 3G) and the KLAL ARK cornea (Fig 3H), and strongly in the centered transplanted ARK cornea (Fig 3I) and decentered transplanted ARK cornea (Fig 3J). Collagen IV was also detected in the blood vessels in all ARK corneas but was absent in control corneas (Fig 3F–3J).

The collagen receptor α11 integrin chain labeled the contours of epithelial cells in both normal (Fig 3K), naïve ARK, KLAL ARK, and centered and decentered transplanted ARK corneas (Fig 3L–3O). It was also seen in patches in the subepithelial pannus in the ARK corneas (Fig 3L–3O).

Laminin α3 chain, normally present in the basement membrane of the corneal epithelium (Fig 3P), was present in disperse streaks in the pannus and in the fragmented epithelial basement membrane in all the aniridia cases (Fig 3Q–3T).

**Fig 4:**
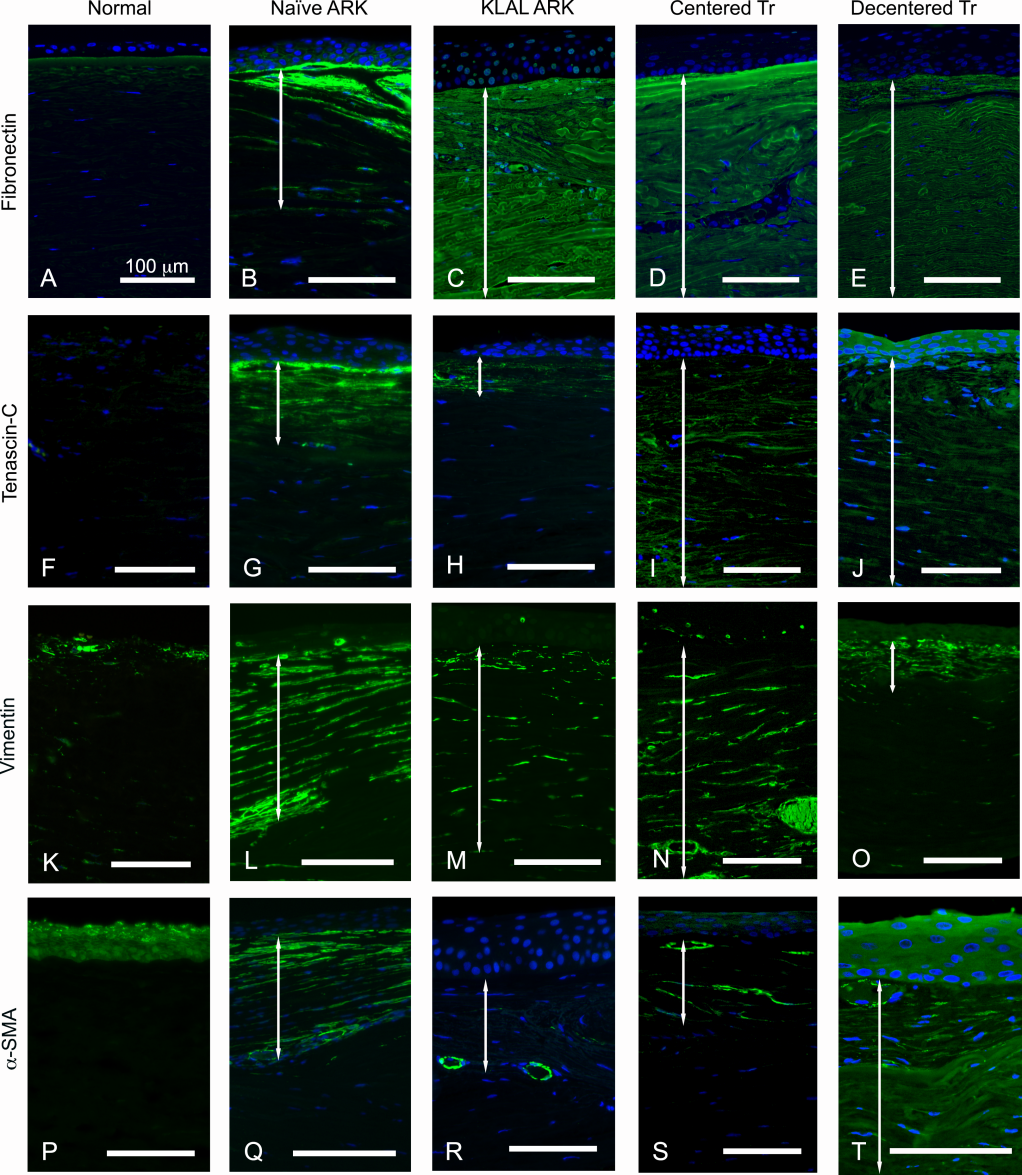
**Cross-sections of normal corneas (A, F, K, P) and naïve ARK corneas (B, G, L, Q), KLAL ARK cornea (C, H, M, R), centered transplanted ARK cornea (D, I, N, S) and decentered transplanted ARK cornea (E, J, O, T) labeled with antibodies (green) against fibronectin (A-E), tenascin-C (F-J), vimentin (K-O),** α**-SMA (P-T).** Cell nuclei are stained blue by DAPI in A-J and Q-T. The double white arrows in B-E, G-J, L-O and Q-T indicate the subepithelial pannus. Fibronectin and tenascin-C play a major role in corneal wound healing and immunolabeling with antibodies against these proteins was present in the pannus (B-E, G-J). The normal cornea (A) displayed sparse fine streaks labelled with the antibody against fibronectin and was not labelled with the antibody against tenascin C (F). An antibody against vimentin labeled keratocytes and patches in the central part of the normal corneal stroma (K). In contrast, the pannus of ARK corneas was strongly labeled with this antibody (L-O). An antibody to α-SMA, myofibroblast marker, did not label the stroma in normal corneas (P). In naïve and decentered transplanted ARK corneas, the subepithelial pannus was strongly labeled (Q and T), whereas in KLAL ARK and centered transplanted ARK corneas, it was only scarcely labelled (R and S), indicating an ongoing wound healing process in ARK corneas (Q-T). The blood vessels in the subepithelial pannus were labeled in all ARK corneas (Q-T). Bars 100 μm.

Weak immunolabeling for fibronectin, a marker of wound healing and fibrosis, was present in sparse fine streaks in the stroma of normal corneas (Fig 4A). In contrast, very strong labeling with this antibody was widely present in the subepithelial pannus in both naïve ARK, KLAL ARK, centered and decentered transplanted ARK corneas (Fig 4B–4E). At higher magnification, the morphology of the lamellae immunolabeled with the antibody against fibronectin was very irregular.

Tenascin-C plays a major role in corneal wound healing and was not present in the normal cornea (Fig 4F), but it was present in streaks in the areas containing subepithelial pannus in all ARK corneas (Fig 4G–4J).

The antibody against vimentin labeled the keratocytes in the normal corneal stroma (Fig 4K). In contrast, strong immunolabeling with this antibody was abundantly present in the pannus of all ARK corneas (Fig 4L–4O).

The subepithelial pannus was strongly labeled with the antibody against α-SMA, a myofibroblast marker, in the naïve and the decentered transplanted ARK corneas (Fig 4Q and 4T), and more scarcely in KLAL ARK and the centered transplanted ARK corneas (Fig 4R and 4S), whereas the stroma of the normal corneas was not labelled (Fig 4P). The blood vessels in the subepithelial pannus were labeled with this antibody in all ARK corneas (Fig 4Q–4T).

**Fig 5:**
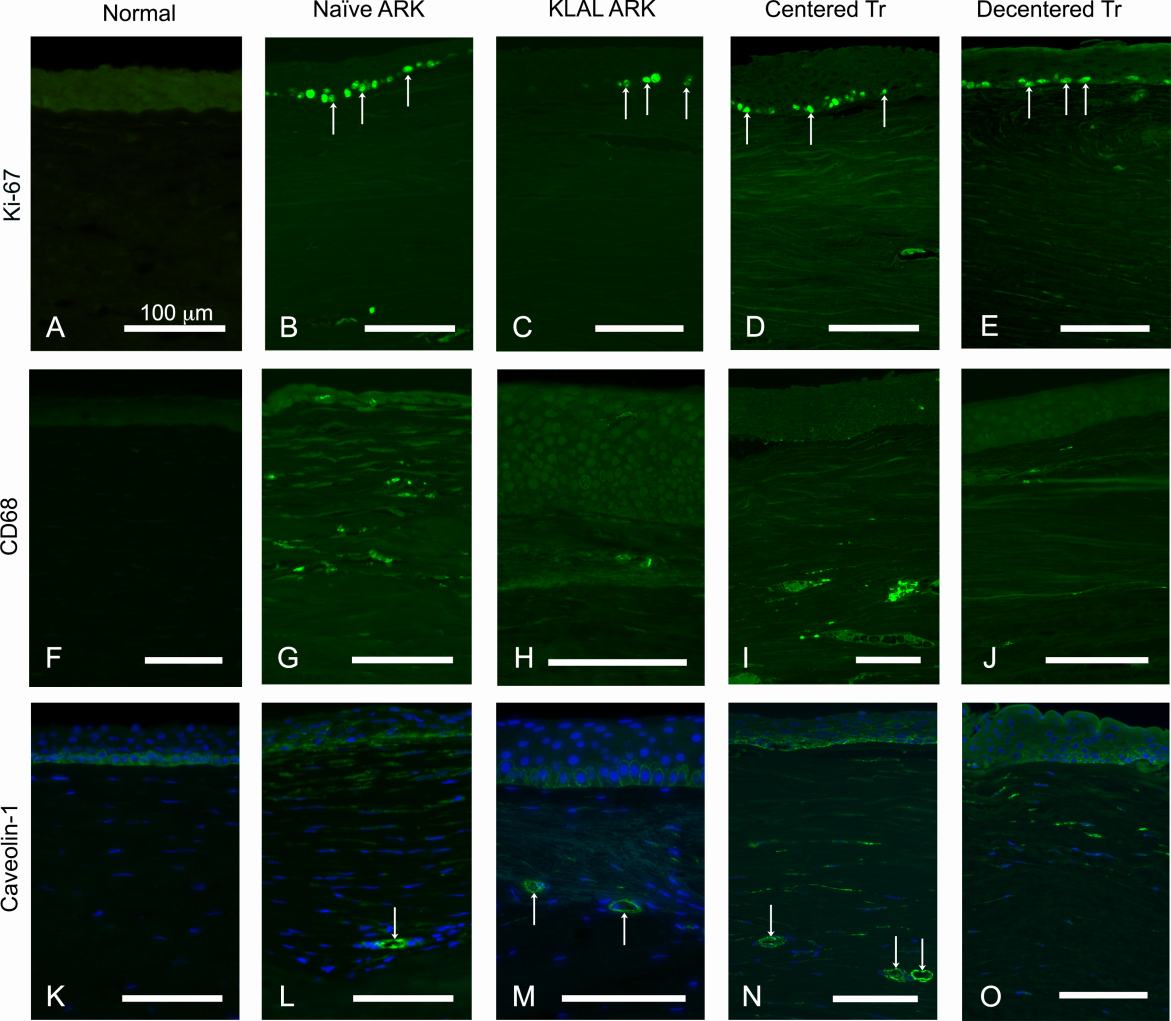
**Cross-sections of normal corneas (A, F, K) and naïve ARK corneas (B, G, L), KLAL ARK cornea (C, H, M), centered transplanted ARK cornea (D, I, N) and decentered transplanted ARK cornea (E, J, O) labeled with antibodies (green) against Ki-67 (A-E), CD68 (F-J) and caveolin-1 (K-O).** Cell nuclei are labeled blue by DAPI in K-O. Ki-67, a marker of cell division, was present in cells of the basal epithelial layer (short arrows) and in sporadic cells in the stroma of both naïve ARK, KLAL ARK, centered and decentered transplanted ARK corneas (B-E), but it was lacking from the central part of the normal control corneas (A). Immunostaining with CD68 was present in sporadic cells in the epithelium and more abundantly in the pannus of both naïve ARK (G), KLAL ARK (H), centered (I) and decentered (J) transplanted ARK corneas. Antibodies against caveolin-1 labeled the basal epithelial cells in control normal corneas (K). Labelling with this antibody was present in the basal epithelial layer in ARK corneas (L-O), as well as in the blood vessels and in streaks in the subepithelial pannus of naïve ARK, KLAL ARK, centered and decentered transplanted ARK corneas, (L-O). Bars 100 μm.

Ki-67, a marker of cell division, was absent from the central part of the normal control corneas (Fig 5A) but in both naïve ARK, KLAL ARK, centered and decentered transplanted ARK corneas it was present in numerous cells in the basal epithelial layer and in sporadic cells in the anterior as well as in the deeper parts of the stroma (Fig 5B–5E).

Immunostaining with CD68, a macrophage marker, was absent in control normal corneas (Fig 5F) but it was sporadically present in the epithelium and more abundantly in the pannus of all ARK corneas (Fig 5G–5J), with no significant differences between the different ARK corneas.

Labeling with antibodies against caveolin-1 was present in the basal epithelial layer in normal corneas (Fig 5K). In ARK corneas, these antibodies also labeled the basal epithelial layer and, in addition, labeled the blood vessels and streaks in the subepithelial pannus (Fig 5L–5O).

## Discussion

This is the first study to investigate both naïve corneas of aniridia patients with severe ARK, an ARK cornea from a patient that had previously undergone a keratolimbal autograft transplant but without success in restoring a normal ocular surface; and transplanted corneal buttons that were initially from healthy donors but had been transplanted to aniridia patients that subsequently developed severe ARK and required re-transplantation. Decentered keratolimbal transplantation is considered to be the best first-line surgical treatment for advanced ARK [5, 11], as centered penetrating keratoplasty alone has a failure rate of 64–100% [33]. KLAL, in particular the limbal allograft transplantation with the modified protocol described by Holland and co-workers [13] is also considered a valid option for patients with corneal pathologies that primarily involve limbal stem cells, such as ARK [15, 16]. Nevertheless, both decentered keratolimbal transplantation and KLAL are also associated with limited success in aniridia, with Boston keratoprosthesis being the last surgical treatment option available. Our samples from failed decentered corneal transplantations and KLAL constitute, therefore, an opportunity to further evaluate the pathophysiology associated with the recurrence of ARK.

The major findings of this study regarding ARK are:

- in all ARK corneas, there was a thin and irregular epithelium and disruption or absence of the epithelial basement membrane and Bowman’s layer.
- in both naïve ARK, KLAL ARK and transplanted ARK corneas there was a subepithelial pannus where collagen I was not present. All ARK corneas showed a loss of the orderly pattern of collagen lamellae.
- there were signs of corneal vascularization revealed by the presence of caveolin-1 and collagen IV in the pannus of both all ARK corneas.
- both naïve, KLAL ARK and transplanted ARK corneas, irrespective of being centered or decentered, presented similar histopathological changes.

This study confirmed previous results concerning the thin and irregular epithelium with disruption to almost total absence of epithelial basement membrane and Bowman’s layer in ARK [3, 34, 35, 36, 37]. The absence of epithelial basement membrane hampers the anchorage of epithelial cells, further aggravating the deficient corneal wound healing [38]. The epithelial basement membrane has a role in determining the levels of certain epithelial proliferation, differentiation and motility modulators that are produced in the stroma, such as keratinocyte growth factor [18, 39]. It also functions as a regulatory structure that limits the stromal fibrotic response by influencing the epithelium-derived levels of PDGF, TGF-ß and possibly other growth factors and extracellular matrix components that can reach stromal cells [40]. Further studies are needed to investigate whether such changes are also part of the pathophysiology of ARK. The antibody against collagen IV did not label the epithelial basement membrane in the normal corneas. This may be due to the fact that the antibody used may merely recognize α1 and α2 chains, which have been shown to be expressed only in the limbus in adult human corneas, but absent from the central part of the cornea [41]. The α3 and α5 chains of collagen IV are specifically expressed in the central part of the cornea [41,42] and we suspect that these were not recognized by the collagen IV antibody used.

In the anterior stroma of the ARK corneas, collagen I was absent and the fibrotic components fibronectin, tenascin-C, vimentin and α-SMA were abundantly present. The absence of collagen I lamellae in the ARK corneas mimics the pattern described in Pax6+/- mice [35] and explains the loss of corneal transparency and impaired vision in these patients. The increased presence of α11 integrin in the pannus regions of the ARK corneas mimics the distribution pattern in the anterior stroma of keratoconus with scarring [21] and is in line with previous reports on the importance of the α11 integrin chain in the remodeling of corneal extracellular matrix and its role in the interaction with collagen I and IV [20]. The presence of vimentin and α-SMA in the pannus identifies myofibroblasts and abnormal extracellular matrix deposition, suggesting ongoing wound healing [25]. Furthermore, α-SMA staining of the subepithelial pannus vessels likely indicates that they are mature, pericyte-covered vessels [43]. The abundant tenascin-C and fibronectin in the fibrotic pannus paralleled the finding of these markers in scarred corneas [44, 45, 46].

The strong and abundant labeling seen with antibodies against Ki-67 indicates ongoing cell proliferation [47]. The present data also suggest ongoing macrophage activity revealed by abundant CD68 labeling in the ARK corneas. CD68 is a pan-macrophage marker. Macrophages can, nevertheless, differentiate to different subsets, with pro (M1) or anti (M2) angiogenic activity [48]. Even though no specific marker for the different subtypes of macrophages was used, given their localization in the subepithelial pannus, we speculate that the main subset of macrophages present is likely to be M1. The staining patterns obtained with the antibodies against Ki-67 and CD68 were rather different and can be interpreted as signs of ongoing cell division mostly in the basal epithelial layer and inflammatory, macrophage-mediated activity more prominently in the subepithelial pannus.

Superficial stromal neovascularization is one of the primary steps in ARK [35] and neovascularization was documented in the pannus of all our cases by the presence of caveolin-1 and collagen IV markers in all ARK corneas.

The best surgical treatment available for severe ARK is penetrating keratoplasty with limbokeratoplasty [5, 11]. Another valid option for treatment is limbal allograft, such as KLAL. Limbal allografting led to corneal reepithelization and improvement of visual acuity [49], emphasizing the importance of limbal stem cell deficiency in the genesis of ARK. KLAL transplantation has been reported to lead to a stable ocular surface in up to 73% of patients with different conditions associated with limbal stem cell deficiency [15, 16].

Healthy donor corneas, after being transplanted to aniridia patients, presented the same histopathological structural changes as naïve ARK corneas, with abnormal epithelium, disruption and/or absence of the epithelial basement membrane, lack of collagen I, fibrotic scarring of the stroma and neovascularization. Limbal stem cell deficiency has been regarded as an important factor in the pathogenesis of ARK, and the existence of a healthy epithelium and epithelial basal membrane appear to be fundamental. The stimulation of keratocytes by epithelial factors and the subsequent associated stromal structural changes have been proposed to be an important part of the pathophysiology of ARK. Limbal allografts have been considered a valid surgical treatment approach in these patients as they would provide a source for limbal stem cells and possible reconstruction of an epithelial barrier. Nevertheless, the KLAL ARK corneal button of the present study mimicked the changes present in the naïve ARK corneas, which suggests either a failure of the limbal allograft transplant in providing a lasting source for limbal stem cells or the existence of an inhospitable corneal microenvironment that did not support these cells or both. After a centered corneal transplantation, all donor epithelial cells are replaced by recipient epithelium as early as within 3 months. Even though a higher proportion of donor stromal cells survive after transplantation, their replacement by the recipient´s own stromal cells does occur to a certain degree [50]. The percentage of recipient stromal keratocytes after transplantation in an ARK patient is, therefore, predicted to be variable and their contribution to the local altered microenvironment might also be a determining factor for the progression of ARK and success or failure of the surgical treatments.

The similar findings between both the KLAL ARK cornea and the decentered keratolimbal transplanted corneal samples with the centered transplanted cornea sample, further raise the question of whether the transplanted limbal stem cells can survive in these patients, on a long-term perspective. The similarity between the KLAL and decentered transplant corneal buttons, which both later had developed ARK, with the corneal button resulting from the centered transplant might result from the absence of viable limbal stem cells in the KLAL and decentered buttons. Their survival might be affected by both the host´s immune response and by the characteristics of the stem cell niche milieu and some authors have suggested that the outcomes for this approach would be improved if a HLA-matched related donor could be used [51]. These findings also stress the need for systemic immunosupressive treatment in limbal stem cell transplantation.

In summary, in all naïve ARK, KLAL ARK and transplanted ARK corneas, we found evidence of abnormal epithelium and basement membrane and similar patterns of derangement of the corneal stromal architecture, with a pannus in the superior part of the stroma lacking collagen I, displaying fibrotic markers and blood vessels. The corneas in the naïve ARK cases were only naïve in the sense that they were not previously submitted to corneal transplantation procedures. It is, therefore, impossible to separate all the histologic changes due to ARK from those expected due to therapeutic ablation or other causes of erosion, healing and fibrosis. The findings of the present study have to be interpreted considering the limitations related to the low number of aniridia cases which are inherent to the scarcity of surgical samples from these patients.

The present results indicate the possible existence of an abnormal microenvironment that is apparently similar in naïve ARK, KLAL ARK, transplanted centered donor corneas and in transplanted decentered donor corneas, suggesting that the conditions of the host play a major role on the corneal features in the long run. Further studies are underway to elucidate the possible mechanisms behind the altered corneal microenvironment in aniridia patients.

## References

[1] Eden U, Beijar C, Riise R, Tornqvist K. Aniridia among children and teenagers in Sweden and Norway. Acta Ophthalmol. 2008; 86:730–734. doi: 10.1111/j.1755-3768.2008.01310.x 18494744 10.1111/j.1755-3768.2008.01310.x

[2] Ihnatko R, Eden U, Fagerholm P, Lagali N. Congenital Aniridia and the Ocular Surface. Ocul Surf. 2016; 14(2):196–206. doi: 10.1016/j.jtos.2015.10.003 26738798 10.1016/j.jtos.2015.10.003

[3] Bausili MM, Alvarez de Toledo J, Barraquer RI, Michael R, Tresserra F, de la Paz MF. Histopathology Findings of Corneal Buttons in Congenital Aniridia Patients. Ophthalmic Res. 2016; 56(4):202–206. doi: 10.1159/000444930 27160090 10.1159/000444930

[4] Auw-Haedrich C, Agrawal M, Gabbert HE, Meyer P, Arnold N, Reinhard T. Immunohistochemical expression of epithelial cell markers in corneas with congenital aniridia and ocular cicatrizing pemphigoid. Acta Ophthalmol. 2011; 89(1):47–53. doi: 10.1111/j.1755-3768.2009.01603.x 19558573 10.1111/j.1755-3768.2009.01603.x

[5] Lee H, Khan R, O'Keefe M. Aniridia: current pathology and management. Acta Ophthalmol. 2008; 86:708–715. doi: 10.1111/j.1755-3768.2008.01427.x 18937825 10.1111/j.1755-3768.2008.01427.x

[6] Kawashima M, Kawakita T, Higa K, Satake Y, Omoto M, Tsubota K, et al Subepithelial corneal fibrosis partially due to epithelial-mesenchymal transition of ocular surface epithelium. Mol Vis. 2010; 16:2727–2732. 21179238 PMC3002964

[7] Tsai JH, Freeman JM, Chan CC, Schwartz GS, Derby EA, Petersen MR, et al A progressive anterior fibrosis syndrome in patients with postsurgical congenital aniridia. Am J Ophthalmol. 2005; 140:1075–1079. doi: 10.1016/j.ajo.2005.07.035 16376654 10.1016/j.ajo.2005.07.035

[8] Eden U, Riise R, Tornqvist K. Corneal involvement in congenital aniridia. Cornea. 2010; 29:1096–1102. doi: 10.1097/ICO.0b013e3181d20493 20567200 10.1097/ICO.0b013e3181d20493

[9] Lagali N, Eden U, Utheim TP, Chen X, Riise R, Dellby A, et al In vivo morphology of the limbal palisades of vogt correlates with progressive stem cell deficiency in aniridia-related keratopathy. Invest Ophthalmol Vis Sci. 2013; 54:5333–5342. doi: 10.1167/iovs.13-11780 23860752 10.1167/iovs.13-11780

[10] Eden U, Fagerholm P, Danyali R, Lagali N. Pathologic epithelial and anterior corneal nerve morphology in early-stage congenital aniridic keratopathy. Ophthalmology. 2012; 119:1803–1810. doi: 10.1016/j.ophtha.2012.02.043 22512983 10.1016/j.ophtha.2012.02.043

[11] Holland EJ, Djalilian AR, Schwartz GS. Management of aniridic keratopathy with keratolimbal allograft: a limbal stem cell transplantation technique. Ophthalmology. 2003; 110:125–130. 12511357 10.1016/s0161-6420(02)01451-3

[12] Tsubota K, Toda I, Saito H, Shinozaki N, Shimazaki J. Reconstruction of the corneal epithelium by limbal allograft transplantation for severe ocular surface disorders. Ophthalmology. 1995; 102:1486–95. 9097796 10.1016/s0161-6420(95)30841-x

[13] Holland EJ. Epithelial transplantation for the management of severe ocular surface disease. Trans Am Ophthalmol Soc. 1996; 19:677–743. PMC1312113 8981714

[14] Holland EJ, Schwartz GS. Changing Concepts in the Management of Severe. Ocular Surface Disease Over Twenty-five Years. Cornea. 2000; 19(5):688–698. 11009321 10.1097/00003226-200009000-00014

[15] Kim JY, Djalilian AR, Schwartz GS, Holland EJ. Ocular surface reconstruction: Limbal stem cell transplantation. Ophthalmol Clin N Am. 2003; 16:67–77. 10.1016/s0896-1549(02)00107-4 12683249

[16] Bakhtiari P, Djalilian A. Update on Limbal Stem Cell Transplantation. Middle East Afr J Ophthalmol. 2010; 17(1): 9–14. doi: 10.4103/0974-9233.61211 20543931 10.4103/0974-9233.61211 PMC2880366

[17] Shepard J, Hayres S, Boote C, Votruba M, Meek K. Changes in Corneal Collagen Architecture during Mouse Postnatal Development. Invest Ophthalmol Vis Sci. 2010; 51(6):2936–42. doi: 10.1167/iovs.09-4612 20089872 10.1167/iovs.09-4612

[18] Ljubimov AV, Saghizadeh M. Progress in corneal wound healing. Prog Retin Eye Res. 2015; 49:17–45. doi: 10.1016/j.preteyeres.2015.07.002 26197361 10.1016/j.preteyeres.2015.07.002 PMC4651844

[19] Ihanamäki T, Pelliniemi LJ, Vuorio E. Collagens and collagen-related matrix components in the human and mouse eye. Prog Retin Eye Res. 2004; 23(4):403–34. doi: 10.1016/j.preteyeres.2004.04.002 15219875 10.1016/j.preteyeres.2004.04.002

[20] Zhang W, Kapyla J, Puranen J. α11β1 integrin recognizes the GFOGER sequence in interstitial collagens. J Biol Chem. 2003; 278:7270–7277. doi: 10.1074/jbc.M210313200 12496264 10.1074/jbc.M210313200

[21] Byström B, Carracedo S, Behndig A, Gullberg D, Pedrosa-Domellöf F. Alpha11 integrin in the human cornea: importance in development and disease. Invest Ophthalmol Vis Sci. 2009; 50(11):5044–53. doi: 10.1167/iovs.08-3261 19516006 10.1167/iovs.08-3261

[22] Saika S, Yamanaka O, Okada Y, Sumioka T. Modulation of Smad signaling by non-TGFβ components in myofibroblast generation during wound healing in corneal stroma. Exp Eye Res. 2016; 142:40–8. doi: 10.1016/j.exer.2014.12.015 26675402 10.1016/j.exer.2014.12.015

[23] Sumioka T, Fujita N, Kitano A, Okada Y, Saika S. Impaired angiogenic response in the cornea of mice lacking tenascin C. Invest Ophthalmol. Vis. Sci. 2011; 16;52(5):2462–7 doi: 10.1167/iovs.10-5750 21087965 10.1167/iovs.10-5750

[24] Nagamoto T, Eguchi G, Beebe DC. Alpha-smooth muscle actin expression incultured lens epithelial cells. Invest Ophthalmol Vis Sci. 2000; 41(5):1122–9. 10752950

[25] Das SK, Gupta I, Cho YK, Zhang X, Uehara H, Muddana SK, et al Vimentin knockdown decreases corneal opacity. Invest Ophthalmol Vis Sci. 2014; 55:4030–4040. doi: 10.1167/iovs.13-13494 24854859 10.1167/iovs.13-13494 PMC4078947

[26] Rhim JH, Kim JH, Yeo EJ, Kim JC, Park SC. Caveolin-1 as a novel indicator of wound-healing capacity in aged human corneal epithelium. Mol Med. 2010; 16:527–534. doi: 10.2119/molmed.2010.00046 20644900 10.2119/molmed.2010.00046 PMC2972400

[27] Armitage WJ, Dick AD, Bourne WM. Predicting endothelial cell loss and long-term corneal graft survival. Invest Ophthalmol Vis Sci. 2003; 44: 3326–3331. 12882777 10.1167/iovs.02-1255

[28] Cornea Donor Study Investigator Group, Gal RL, Dontchev M, Beck RW, Mannis MJ, Holland EJ, et al The effect of donor age on corneal transplantation outcome results of the cornea donor study. Ophthalmology. 2008; 115:620–626.e6. doi: 10.1016/j.ophtha.2008.01.003 18387407 10.1016/j.ophtha.2008.01.003 PMC2810523

[29] Ramaesh T, Collinson JM, Ramaesh K, Kaufman MH, West JD, Dhillon B. Corneal abnormalities in Pax6+/- small eye mice mimic human aniridia-related keratopathy. Invest Ophthalmol Vis Sci. 2003; 44:1871–1878. 12714618 10.1167/iovs.02-0576

[30] Robertson D, Savage K, Reis-Filho JS, Isacke CM. Multiple immunofluorescence labelling of formalin-fixed paraffin-embedded (FFPE) tissue. BMC Cell Biol. 2008; 19:9–13. 10.1186/1471-2121-9-13 PMC2288605 18366689

[31] Byström B, Virtanen I, Rousselle P, Miyazaki K, Lindén C, Pedrosa Domellöf F. Laminins in normal, keratoconus, bullous keratopathy and scarred human corneas. Histochem Cell Biol. 2007; 127:657–667. doi: 10.1007/s00418-007-0288-4 17492460 10.1007/s00418-007-0288-4

[32] Byström B, Virtanen I, Rousselle P, Gullberg D, Pedrosa Domellöf F. Distribution of Laminins in the Developing Human Eye. Invest Ophthalmol Vis Sci. 2006; 47:777–785. doi: 10.1167/iovs.05-0367 16505007 10.1167/iovs.05-0367

[33] Lim HT, Kim DH, Kim H. PAX6 aniridia syndrome: clinics, genetics, and therapeutics. Curr Opin Ophthalmol. 2017; 28(5):436–447 doi: 10.1097/ICU.0000000000000405 28598868 10.1097/ICU.0000000000000405

[34] Kawashima M, Kawakita T, Higa K, Satake Y, Omoto M, Tsubota K, et al Subepithelial corneal fibrosis partially due to epithelial-mesenchymal transition of ocular surface epithelium. Mol Vis. 2010; 16:2727–2732. 21179238 PMC3002964

[35] Ramaesh T, Ramaesh K, Martin Collinson J, Chanas SA, Dhillon B, West JD. Developmental and cellular factors underlying corneal epithelial dysgenesis in the Pax6+/- mouse model of aniridia. Exp Eye Res. 2005; 81:224–235. doi: 10.1016/j.exer.2005.02.002 16080917 10.1016/j.exer.2005.02.002

[36] Espana EM, Di Pascuale MA, He H, Kawakita T, Raju VK, Liu CY, et al Characterization of corneal pannus removed from patients with total limbal stem cell deficiency. Invest Ophthalmol Vis Sci. 2004; 45:2961–2966. doi: 10.1167/iovs.03-1397 15326108 10.1167/iovs.03-1397

[37] Le Q, Deng SX, Xu J. In vivo confocal microscopy of congenital aniridia-associated keratopathy. Eye. 2013; 27:763–766. doi: 10.1038/eye.2013.50 23579408 10.1038/eye.2013.50 PMC3682356

[38] Torricelli AA, Singh V, Santhiago MR, Wilson SE. The Corneal Epithelial Basement Membrane: Structure, Function, and Disease. Invest Ophthalmol Vis Sci. 2013; 54:6390–6400. doi: 10.1167/iovs.13-12547 24078382 10.1167/iovs.13-12547 PMC3787659

[39] Wilson SE, He YG, Weng J, Zieske JD, Jester JV, Schultz GS. Effect of epidermal growth factor, hepatocyte growth factor, and keratinocyte growth factor, on proliferation, motility and differentiation of human corneal epithelial cells. Exp Eye Res. 1994; 59:665–678. doi: 10.1006/exer.1994.1152 7698260 10.1006/exer.1994.1152

[40] Singh V, Agrawal V, Santhiago MR, Wilson SE. Stromal fibroblast-bone marrow-derived cell interactions: implications for myofibroblast development in the cornea. Exp Eye Res. 2012; 98:1–8. doi: 10.1016/j.exer.2012.03.006 22465408 10.1016/j.exer.2012.03.006 PMC3340470

[41] Ljubimov AV, Burgeson RE, Butkowski RJ, Michael AF, Sun TT, Kenney MC. Human corneal basement membrane heterogeneity: topographical differences in the expression of type IV collagen and laminin isoforms. Lab Invest. 1995; 72(4):461–73. 7723285

[42] Ljubimov AV, Alba SA, Burgeson RE, Ninomiya Y, Sado Y, Sun TT, et al Extracellular matrix changes in human corneas after radial keratotomy. Exp Eye Res. 1998; 67(3):265–72. doi: 10.1006/exer.1998.0511 9778407 10.1006/exer.1998.0511

[43] Gerhardt H., Betsholtz C. Endothelial-pericyte interactions in angiogenesis. Cell Tissue Res. 2003; 314, 15e23. 12883993 10.1007/s00441-003-0745-x

[44] Tuori A, Virtanen I, Aine E, Uusitalo H. The expression of tenascin and fibronectin in keratoconus, scarred and normal human cornea. Graefes Arch Clin Exp Ophthalmol. 1997; 235:222–229. 9143890 10.1007/BF00941763

[45] Hsu J, Rubinfeld R, Barry P, Jester J. Anterior stromal puncture. Immunohistochemical studies in human corneas. Arch Ophthalmol. 1993; 111:1057–1063. 8352688 10.1001/archopht.1993.01090080053018

[46] Karamichos D, Guo XQ, Hutcheon AE, Zieske JD. Human corneal fibrosis: an in vitro model. Invest Ophthalmol Vis Sci. 2010; 51:1382–1388. doi: 10.1167/iovs.09-3860 19875671 10.1167/iovs.09-3860 PMC2868432

[47] Torp SH. Proliferative activity in human glioblastomas: evaluation of different Ki67 equivalent antibodies. Mol Pathol. 1997; 50:198–200. 9350303 10.1136/mp.50.4.198 PMC379626

[48] Mosser DM, Edwards JP. Exploring the full spectrum of macrophage activation. Nat Rev Immunol. 2008; 8:958–969. doi: 10.1038/nri2448 19029990 10.1038/nri2448 PMC2724991

[49] de la Paz MF, Alvarez de Toledo J, Barraquer RI, Barraquer J. Long-term visual prognosis of corneal and ocular surface surgery in patients with congenital aniridia. Acta Ophthalmol. 2008; 86(7):735–40. doi: 10.1111/j.1755-3768.2008.01293.x 18631333 10.1111/j.1755-3768.2008.01293.x

[50] Lagali N, Stenevi U, Claesson M, Fagerholm P, Hanson C, Weijdegård B, et al Survival of donor-derived cells in human corneal transplants. Invest Ophthalmol Vis Sci. 2009; 50(6):2673–8. doi: 10.1167/iovs.08-2923 19151390 10.1167/iovs.08-2923

[51] Reinhard T, Spelsberg H, Henke L, Kontopoulos T, Enczmann J, Wernet P, et al Long-term results of allogeneic penetrating limbo-keratoplasty in total limbal stem cell deficiency. Ophthalmology. 2004; 111(4):775–82. doi: 10.1016/j.ophtha.2003.07.013 15051212 10.1016/j.ophtha.2003.07.013

